# The Inspiration of Example

**Published:** 1921-09-17

**Authors:** 


### The Inspiration of Example.

THE retirement from office of three noted matrons before the autumn session begins forces upon the mind reflections of the unique powers and influence possessed by the heads of the nurse training-schools in the present day. Miss Janet Melrose, who received warm tokens of the affection with which her staff regard her at the farewell gathering held on August 29, numbers thirty years' devoted service at the Royal Infirmary, Glasgow, and has done much which will leave its mark on the profession at large. Miss Deane of the East Suffolk and Ipswich Hospital, has spent in all thirty-five years of her life in the institution, and is greatly beloved there. It is hard to imagine it without her sustaining presence. Miss Crookenden, in comparison with these veteran matrons, may be reckoned a comparatively new holder of office at Addenbrooke's Hospital, Cambridge, but during the last eight difficult years she has profoundly influenced the training-school, and her resignation has roused deep regret among all who have had the privilege of working under her.

We doubt if there were ever a time when the matrons of institutions were as truly "autocratic" as they are painted in the public imagination. Certainly for many years past the position has been one which is hemmed about with many restrictions and demands the exercise of any but "autocratic" qualities. The matron to be successful must work harmoniously with her Board, with the medical officers and secretary superintendent, with her own assistants, the ward sisters and other heads of departments, must understand the secret of turning raw materials into good nurses, must be able to attract and retain the loyal service of the domestic staff. In every act of her life, that is to say, she is hemmed about by erring mortals, must make the best of them, be ready to resign her own wishes wherever principle is not involved, and yet, firm as a rock, be able to retain the headship of her domain. Autocracy under such conditions would crumble to pieces before the end of a month in office.

But if her prerogative be strictly limited the influence of the matron is practically boundless. We know of no position to which a woman can aspire so well calculated to give scope for her powers and enlarge her character as the direction of a training-school for nurses. In the fierce light which beats upon her office it is possible her little failings may stand out for the petty-minded to criticise. But the propulsive force of a high example sweeps all before it. The self-forgetful purpose of the true matron, mother of her flock, multiplies itself a thousandfold among those she governs, and generations of nurses yet to be will be moulded in accordance with the principles well and soundly laid in her training-school.

Matronship can never be other than a heavy burden however priceless a privilege. To those now laying down the work which has crowned their nursing labours we offer in all respect congratulations upon their success, and on the end of their term of office.

